# Depression, anxiety and stress among Swedish university students during the second and third waves of COVID-19: A cohort study

**DOI:** 10.1177/14034948211031402

**Published:** 2021-07-24

**Authors:** Fred Johansson, Pierre Côté, Sheilah Hogg-Johnson, Eva Skillgate

**Affiliations:** 1 Musculoskeletal & Sports Injury Epidemiology Center, Department of Health Promotion Science, Sophiahemmet University, Sweden; 2 Unit of Intervention and Implementation Research for Worker Health, Institute of Environmental Medicine, Karolinska Institutet, Sweden; 3 Faculty of Health Sciences and Centre for Disability Prevention and Rehabilitation, Ontario Tech University, Canada; 4 Canadian Memorial Chiropractic College, Dalla Lana School of Public Health, University of Toronto, Canada

**Keywords:** Depression, anxiety, stress, mental health, COVID-19, coronavirus, students, Sweden

## Abstract

Our research group recently reported that symptom levels of depression, anxiety, and stress among Swedish university students were relatively stable during the first three months of COVID-19 and decreased slightly during the following three summer months. Since then, Sweden has faced a second and third wave of COVID-19.

**Aims:**

This study aims to describe the mean trajectories of depression, anxiety and stress symptoms among Swedish university students before and during the second and third waves of the COVID-19 pandemic.

**Methods:**

We recruited 1835 participants in September 2020, of whom 81% provided follow-ups in December 2020–January 2021 and 77% provided follow-ups in March–April 2021. The short-form Depression, Anxiety and Stress Scale was used to measure mental health symptoms. Generalized estimating equations were used to estimate the mean differences in symptom levels over the three time periods.

**Results:**

Compared with September, mean depression was 0.91 points of 21 higher (95% confidence interval (CI) 0.70–1.13) in December 2020–January 2021 and 0.66 points higher (95% CI 0.43–.88) in March–April 2021. Anxiety levels were 0.20 points higher (95% CI 0.05–0.34) in December 2020–January 2021 and 0.17 points higher (95% CI 0.02–0.33) in March–April 2021. Stress levels were 0.21 points higher (95% CI 0.00–0.41) in December 2020–January 2021 and 0.16 points lower (95% CI −0.38 to 0.05) in March–April 2021.

**Conclusions:**

**Our results indicate relatively stable levels of mental health among Swedish university students during the second and third waves of COVID-19 compared with before the second wave. Mean depression symptom scores increased slightly, but the importance of this small increase is uncertain.**

## Introduction

Online education became the main form of education in Swedish universities on 17 March 2020, with no or minimal campus-based education since that date. Our research group recently published results from a cohort study describing the trajectories of mental health symptoms among Swedish university students before and during the first six months of the COVID-19 pandemic [1]. We observed stable symptom levels of depression, anxiety and stress during the first wave of the pandemic and a slight decrease in the following summer months.

A second wave of COVID-19 swept over Europe during the autumn and winter of 2020. In Sweden, the number of COVID-19 cases and deaths began to rise markedly in October 2020 and continued rising through November and most of December, with decreasing numbers in late December 2020 and early January 2021 [2]. In March 2021, the spread was once again accelerating [2] into what has been referred to as the third wave. Sweden’s public health response during the first wave of COVID-19 was less restrictive than most other countries [3].

The second wave of the pandemic led to stricter restrictions and recommendations to further reduce physical contact between people [4]. For university students, the most noticeable restriction was the prolonged use of online education as the main form of education, with some exceptions for practical training, such as the clinical rotations of medical students.

The Swedish Higher Education Authority reports that recommendations to avoid travelling and the governmental decision to limit public gatherings to a maximum of eight people has further impacted university students [5,6]. Given the prolonged and more restrictive recommendations, the mental health impact may shift over the course of the pandemic. No study has to our knowledge evaluated the mental health of students during the second and third waves of COVID-19 in Sweden.

This study aimed to describe mean trajectories of depression, anxiety and stress symptoms among Swedish university students before and during the second and third waves of the COVID-19 pandemic.

## Methods

### Design and study population

This study is nested within a larger ongoing dynamic longitudinal cohort study of university students, the Sustainable University Life study (SUN-study) (http://clinicaltrials.gov/, ID NCT04465435), which includes full-time undergraduate students with at least one year left of their education and follows them every three months over one year with web-based surveys. The targeted universities constitute a convenience sample, with the aim of representing a variety of different university programmes. This study uses data from participants entering the SUN-study in September 2020, recruited from seven universities in and around Stockholm and covering medical, technical and social sciences. Online education has been the main educational form at all the universities, although the medical students’ practical education and clinical rotations have been held on campus in smaller groups or at hospitals.

### Data collection

Invited students were informed about the study through online presentations by the study staff and/or received a link to the web-based survey by email. Information about the study was also distributed through social media channels. The responding students filled out their baseline survey in September 2020, their first follow-up survey in December 2020–January 2021 and their second follow-up in March–April 2021. The study was approved by the Swedish Ethical Review Authority (2019-03276, 2020-01449); all participants provided informed consent.

### Outcomes

Symptom levels of depression, anxiety and stress were measured using the short-form Depression, Anxiety, and Stress Scale (DASS-21) at baseline and follow-up. DASS-21 contains 21 items, scored from 0 (‘did not apply to me at all’) to 3 (‘applied to me very much, or most of the time’), covering three subscales: depression, anxiety and stress. Each subscale comprises seven items that are summed giving subscale scores ranging from 0 to 21. The DASS-21 subscales had Cronbach’s α values of 0.77–0.90 at baseline and have previously demonstrated convergent validity among Swedish university students [7].

### Statistical analyses

The statistical analyses are analogous to the method used in our previous report [1]. Generalized estimating equations (GEEs) were used to model the mean levels of mental health symptoms during the three time periods. GEEs provide estimates of the marginal population mean of the outcome, accounting for within-subject correlation over time. Three models were constructed, one for each subscale of DASS-21. As we were interested in the overall mean, only the time period was included as a predictor in the models. The models used robust sandwich estimators, exchangeable working correlation structures and a gaussian link function. Sensitivity analyses with only the participants answering at all three time points were conducted. Supplemental analyses stratified by gender and type of education were conducted by including interaction terms between strata and time.

## Results

In September 2020, 11,761 students were invited to the study and 1835 (16%) agreed to participate. Of those, 81% (*n* = 1490) provided follow-up assessments in December 2020–January 2021 and 77 % (*n* = 1411) in March–April 2021. The sample baseline characteristics are presented in Table I.

**Table I.** Baseline characteristics of the study population.

	Included participants (*n* = 1835)
Age (years)	23.2 ± 5.0
Female sex	1010 (55)
DASS-21 depression score	4.87 ± 4.69
DASS-21 anxiety score	2.98 ± 3.28
DASS-21 stress score	6.31 ± 4.57
Place of origin	
Sweden	1364 (74)
Other Nordic countries	37 (2)
Europe	148 (8)
Outside Europe	286 (16)
Type of study	
Technology	1270 (69)
Medicine	288 (16)
Social sciences	277 (15)
Year of education	
1st	795 (43)
2nd	330 (18)
3rd	260 (14)
4th+	450 (25)

Data presented as mean ± SD values or *n* (%).

**Figure 1.**
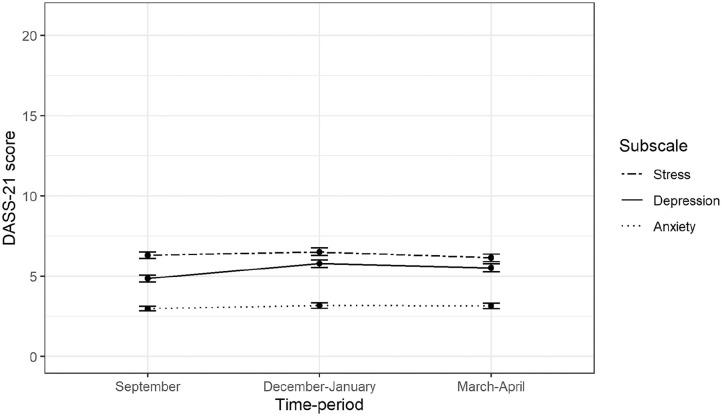
Estimated mean scores on the Depression, Anxiety and Stress Scale (DASS-21) subscales over the three time periods.

Compared with September, mean depression was 0.91 of 21 points higher (95% confidence interval (CI) 0.70–1.13) in December 2020–January 2021 and 0.66 points higher (95% CI 0.43–0.88) in March–April 2021. Anxiety levels were 0.20 of 21 points higher (95% CI 0.05–0.34) in December 2020–January 2021 and 0.17 points higher (95% CI 0.02–0.33) in March–April 2021, compared with September 2020. Stress levels were 0.21 points higher (95% CI 0.00–0.41) in December 2020–January 2021 and were 0.16 points lower (95% CI −0.38 to 0.05) in March–April 2021, compared with September 2020 (Figure 1, Supplemental eTable 1). Results from the complete case sensitivity analyses (*n* = 1324) were all within 0.05 points of the estimates in the main analyses.

The results from the analyses stratified by gender and type of education show similar trajectories across genders and types of education (Supplemental eFigures 1 and 2, Supplemental eTable 1).

## Discussion

We included 1835 students in September 2020, before the second wave of COVID-19 started in Sweden, with follow-ups in December 2020–January 2021 and in March–April 2021. Our results suggest that mean symptom levels of depression were slightly higher during the second and third waves of the COVID-19 pandemic compared with before the second wave. Mean levels of anxiety and stress changed very little during the second and third waves of the pandemic compared with September.

The results of this analysis are consistent with the results of our previous report [1], suggesting that levels of depression, anxiety and stress among Swedish university students have been relatively stable during the COVID-19 pandemic. The slight increase in levels of depression may partly be explained by seasonal variations in mood – the same pattern of increasing depression levels and stable anxiety and stress levels was seen in participants of this cohort followed during the autumn and winter of 2019 before the outbreak of COVID-19 [1]. Alternatively, depression symptoms may be more affected by an increased spread of COVID-19 than anxiety and stress symptoms. This has, however, not been consistently seen in previous research [8].

Most, but not all, previous research on university students during the COVID-19 pandemic has shown larger increases in mental health problems than this study [8]. However, these earlier studies were performed during earlier phases of the pandemic and in countries that implemented different public health strategies than Sweden.

A strength of this study is the large sample of students from seven universities around Stockholm. The high follow-up rate, together with the results from the complete case sensitivity analyses, suggest that attrition bias may have had a minimal effect on our estimates. Because only 16 % of the invited students agreed to participate, and most were technology students, our sample is not demographically representative of Swedish university students overall. However, the supplementary analyses show small differences between the genders and types of education and the trajectories are similar to those of our previous study, which had a different demographic profile. This indicates that our results may be generalized more broadly to Swedish university students.

Our results indicate relatively stable levels of depression, anxiety and stress among Swedish university students during the second and third waves of COVID-19 compared with just before the second wave. Although still small, depressive symptoms scores showed a more pronounced increase compared with the minimal changes in anxiety and stress, but the importance of this small increase is uncertain.

## Supplemental Material

sj-docx-3-sjp-10.1177_14034948211031402 – Supplemental material for Depression, anxiety and stress among Swedish university students during the second and third waves of COVID-19: A cohort study Click here for additional data file. Supplemental material, sj-docx-3-sjp-10.1177_14034948211031402 for Depression, anxiety and stress among Swedish university students during the second and third waves of COVID-19: A cohort study by Fred Johansson, Pierre Côté, Sheilah Hogg-Johnson and Eva Skillgate in Scandinavian Journal of Public Health

sj-JPG-1-sjp-10.1177_14034948211031402 – Supplemental material for Depression, anxiety and stress among Swedish university students during the second and third waves of COVID-19: A cohort study Click here for additional data file. Supplemental material, sj-JPG-1-sjp-10.1177_14034948211031402 for Depression, anxiety and stress among Swedish university students during the second and third waves of COVID-19: A cohort study by Fred Johansson, Pierre Côté, Sheilah Hogg-Johnson and Eva Skillgate in Scandinavian Journal of Public Health

sj-JPG-2-sjp-10.1177_14034948211031402 – Supplemental material for Depression, anxiety and stress among Swedish university students during the second and third waves of COVID-19: A cohort study Click here for additional data file. Supplemental material, sj-JPG-2-sjp-10.1177_14034948211031402 for Depression, anxiety and stress among Swedish university students during the second and third waves of COVID-19: A cohort study by Fred Johansson, Pierre Côté, Sheilah Hogg-Johnson and Eva Skillgate in Scandinavian Journal of Public Health
