# Supplementary material for: Depression, anxiety and stress among Swedish university students during the second and third waves of COVID-19: A cohort study
Source: Scand J Public Health. 2021 Jul 24;49(7):750–4. doi: 10.1177/14034948211031402 (PMC8521365; doi:10.1177/14034948211031402)
Supplement: sj-docx-3-sjp-10.1177_14034948211031402 – Supplemental material for Depression, anxiety and stress among Swedish university students during the second and third waves of COVID-19: A cohort study [file sj-docx-3-sjp-10.1177_14034948211031402.docx]

**eTable 1.** Coefficients from GEE-models of the means of each subscale the depression, anxiety and stress scale (DASS-21) over time for. Both for the main analyses and for the models stratified by gender and type of education.

|  | Depression Coefficients  (95% CI) | Anxiety Coefficients  (95% CI) | Stress Coefficients  (95% CI) |
| --- | --- | --- | --- |
| *Model with only time-period (main analysis)*  Intercept  FU1  FU2 | 4.87 (4.65 to 5.08)  0.91 (0.70 to 1.13)  0.66 (0.43 to 0.88) | 2.98 (2.83 to 3.13)  0.20 (0.05 to 0.34)  0.17 (0.02 to 0.33) | 6.31 (6.10 to 6.52)  0.21 (0.00 to 0.41)  -0.16 (-0.38 to 0.05) |
| *Model with gender and time-period*  Intercept  Gender Male  FU1  FU2  Gender Male * FU1  Gender Male * FU2 | 5.08 (4.78 to 5.37)  - 0.48 (-0.91 to -0.04)  0.99 (0.69 to 1.30)  0.78 (0.46 to 1.09)  -0.19 (-0.61 to 0.24)  -0.29 (-0.74 to 0.16) | 3.51 (3.29 to 3.73)  -1.21 (-1.50 to -0.92)  0.19 (-0.02 to 0.40)  0.12 (-0.11 to 0.35)  -0.00 (-0.29 to 0.29) 0.10 (-0.21 to 0.40) | 7.34 (7.05 to 7.63)  -2.32 (-2.72 to -1.92)  0.15 (-0.13 to 0.44)  -0.24 (-0.56 to 0.07) 0.10 (-0.30 to 0.50)  0.16 (-0.27 to 0.59) |
| *Model with TOE and time -period*  Intercept  Type of education  Technical  Medical  Social sciences  FU1  FU2  TOE:Tech * FU1  TOE:Med *FU1  TOE:SS*FU1  TOE:Tech * FU2  TOE:Med *FU2  TOE:SS *FU2 | 5.08 (4.82 to 5.34)    Ref.  -0.77 (-1.39 to -0.15)  -0.62 (-1.20 to -0.04)  0.76 (0.51 to 1.01)  0.52 (0.25 to 0.80)  Ref.  0.34 (-0.24 to 0.92)  0.65 (-0.00 to 1.29) Ref.  0.37 (-0.21 to 0.95)  0.50 (-0.14 to 1.15) | 2.97 (2.79 to 3.15)    Ref.  0.10 (-0.08 to 0.28)  -0.04 (-0.47 to 0.38) 0.09 (-0.07 to 0.26)  0.10 (-0.08 to 0.28)  Ref.  0.14 (-0.30 to 0.57)  0.54 (0.09 to 1.00)  Ref.  -0.01 (-0.45 to 0.43)  0.49 (0.02 to 0.96) | 6.29 (6.03 to 6.54)    Ref.  0.02 (-0.57 to 0.61)  0.15 (-0.43 to 0.73)  0.05 (-0.19 to 0.29)  -0.22 (-0.48 to 0.03)  Ref.  0.42 (-0.13 to 0.96)  0.56 (-0.05 to 1.17)  Ref.  0.20 (-0.42 to 0.81)  0.19 (-0.42 to 0.80) |

TOE, Type of education; Tech, Technical; Med, Medical; SS, Social sciences; FU1, follow-up period 1 (December-January); FU2, follow-up period 2 (March-April). Models with gender are performed only on participants answering that they are either male or female (n=1825), female is the reference category. In models stratified by type of education Technical is the reference category.
