# Supplementary figures and images for: Depression, anxiety and stress among Swedish university students during the second and third waves of COVID-19: A cohort study
Source: Scand J Public Health. 2021 Jul 24;49(7):750–4. doi: 10.1177/14034948211031402 (PMC8521365; doi:10.1177/14034948211031402)

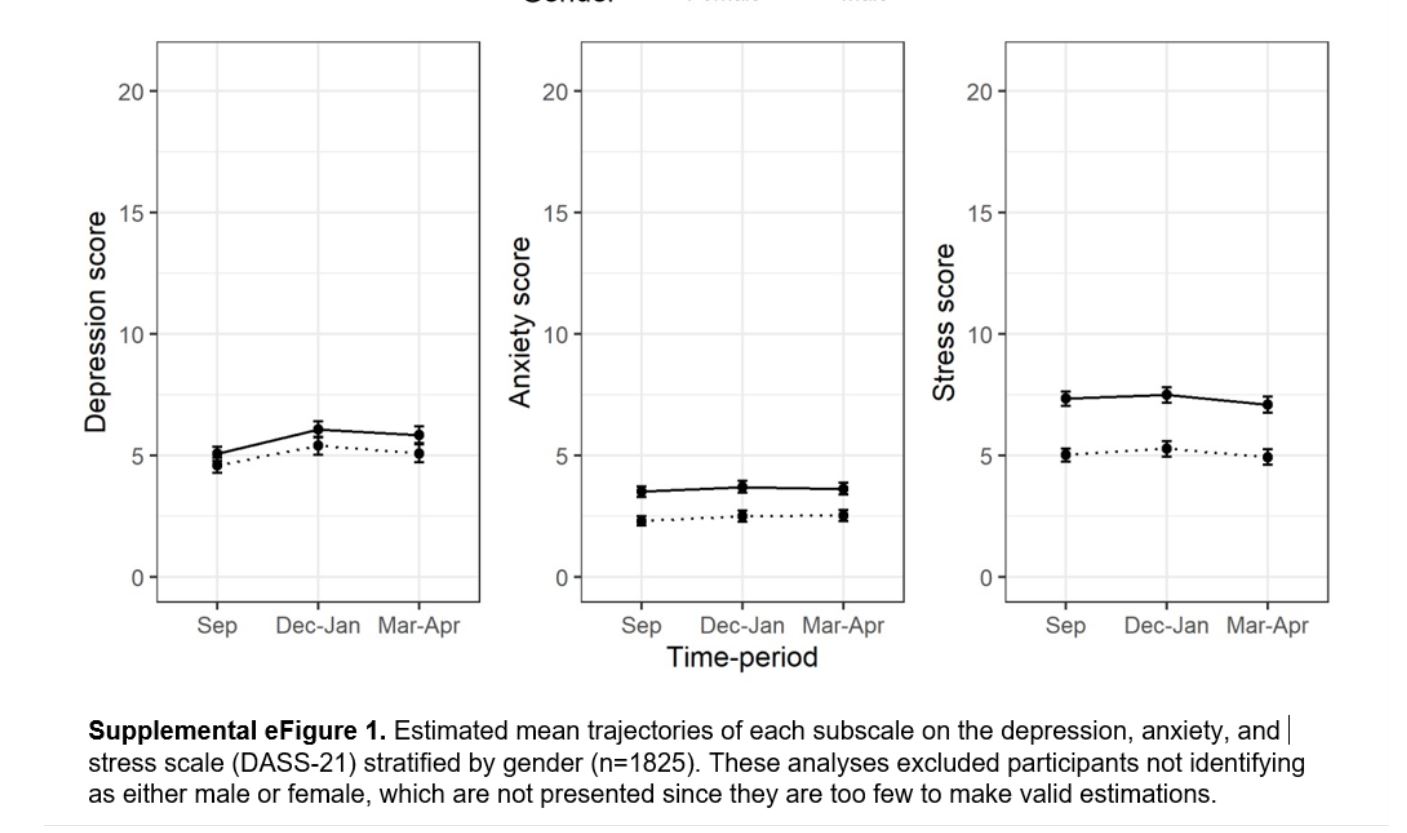

Supplement: sj-JPG-1-sjp-10.1177_14034948211031402 – Supplemental material for Depression, anxiety and stress among Swedish university students during the second and third waves of COVID-19: A cohort study [file sj-JPG-1-sjp-10.1177_14034948211031402.JPG]

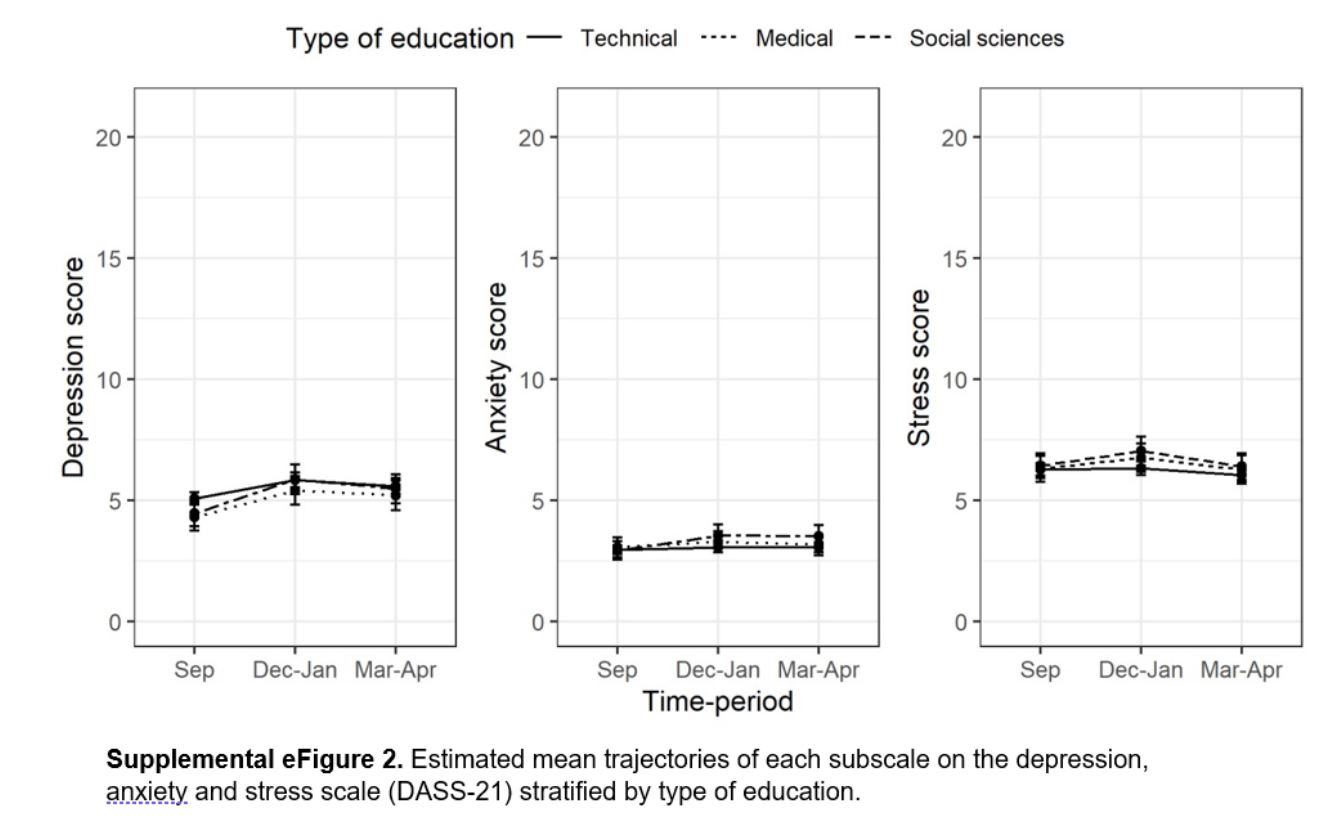

Supplement: sj-JPG-2-sjp-10.1177_14034948211031402 – Supplemental material for Depression, anxiety and stress among Swedish university students during the second and third waves of COVID-19: A cohort study [file sj-JPG-2-sjp-10.1177_14034948211031402.JPG]
